# Drs. Robertson and Ramsay

**Published:** 1851-12

**Authors:** 


*Drs. Robertson and Ramsay.*—The following communications relating to the points at issue between the two gentlemen whose names head the article, have been transmitted to us for publication. The personal difficulties, it appears, are amicably arranged:

*A Card.*—Since the publication of my reply to the pamphlet recently issued by Dr. F. M. Robertson, of Charleston, S. C., I have been in correspondence with Dr. J. J. Robertson, of Washington, Ga., through a friend and having received from him the grounds upon which the opinion of himself and Dr. D. M. Andrews, in regard to my professional statistics was based —which opinion led them when called on by members of the American Medical Association, at their late Convention in Charleston, to express a want of confidence in the reliability of my obstetrical statistics, and induced Dr. F. M. Robertson, a member of that Association, to move to strike out the notice of said statistics from the Report of Dr. Storer, of Boston—and having received from Drs. Andrews and Robertson the assurance that those opinions were neither formed or expressed through personal ill-will towards myself, but were honestly entertained and given in pursuance of what they regarded their duty to the profession; and feeling convinced from the representations of those gentlemen that they were influenced by circumstances, which, unexplained, were calculated to impair their confidence in me as a reporter, I feel bound, in justice to them, to state that, with such impressions on their minds, they could not, when called on, have given other than an unfavorable opinion, and that Dr. F. M. Robertson, in making the motion he did, under the circumstances, was actuated by no unworthy or improper motive.

The grounds on which Drs. Andrews and J. J. Robertson were led to the statement that I had not, during my practice of physic, been continuous in one place, were my removal from Bookersville to Mrs. Wellborn's, a distance of six miles, and subsequently to this place, a distance of one and a half miles, while I regarded myself as never having left the *neighborhood* in which I originally settled. I have also been satisfied, from the statements and evidence furnished me by Dr. J. J. Robertson, that although he has not had a general practice in my neighborhood, he has made over twenty professional visits in the vicinity of my location within the past six years, a fact of which I had not been apprised at the time my late pamphlet was published.

In issuing this card to the profession and the public, I am impelled by no other than a desire to render strict justice to all the parties concerned, and therefore feel bound, under the circumstances, as an honorable man, to withdraw all offensive language I may have used in my pamphlet, or elsewhere, touching this controversy, either towards Drs. Robertson and Andrews, or towards Dr. F. M. Robertson, of Charleston.

I respectfully submit this statement to the profession and the public, with the single additional remark, that it is regarded by all the parties as an amicable and honorable termination of this entire controversy.

H. A. Ramsay.

*Raysville Ga., Oct. 14th.* 1851.

---

*Charleston, S. C., Oct. 31st*, 1851.

Dear Doctor, — Since you handed me the two letters of Dr. H. A. Ramsay to yourself — one dated Sept. 1st, the other Oct. 1st, I have received "a card" — issued by the same gentleman — dated Raysville, Ga., Oct. 14th, 1851, one of which I presume has been sent to you also.

As circumstances growing out of Dr. Ramsay's published defence of August last, and his private correspondence with numerous persons, have drawn me into personal issues with certain parties, which are yet unadjusted, I deem it but just to Dr. Ramsay, as well as due to my own position, to make the following remarks, which will prevent misunderstanding in future.

1st. The card alluded to, was unsolicited by me, but voluntarily tendered by Dr. Ramsay. Nor was the disclaimer, of its author, of imputing "*unworthy or improper motives*" to me, for the course which I thought it my duty to pursue in relation to his obstetrical statistics, or the withdrawal of "*all offensive language in his pamphlet or elsewhere*," in relation to myself, the result of any correspondence or explanation on my part, either *directly* or *indirectly*.

2. Desiring still, as stated in my note to you, of the 28th June, published in my circular of July last, to "throw no impediment in the way of his doing himself ample justice in the matter complained of," I now accept the card as an honorable amend for the imputations against my motives, and his personal abuse, and consider it, so far as I am concerned, a final termination of the personal difficulty between Dr. Ramsay and myself.

3d. Inasmuch as the collateral issues in which I have been involved, (by no act of my own, however) with the editors of certain medical periodicals, in relation to the merits of the "Obstetrical Statistics," are yet unsettled, I hold myself perfectly free, until they are satisfactorily arranged — in case it becomes necessary to my defence — to discuss the professional merits of the points of difference, without its being considered a violation of my acceptance of Dr. Ramsay's card.

4th. If the question should be introduced into the American Medical Association, at its next meeting, which has been intimated from certain quarters, I reserve to myself the same privileges claimed in the preceding paragraph.

Having thus set forth my position, that it may be clearly understood by all parties,

I remain very truly and sincerely,

Your obedient servant,

F. M. Robertson.

Dr. H. W. DeSaussure,
Sec'y A. M. A., Charleston, S. C.

